# Harpers’ Magazine

**Published:** 1863-10

**Authors:** 


*Harpers' Magazine.*—The number for October is a very entertaining one. It commences with an account of the "*First Cruise of the Monitor Passaic,*" which cannot fail to interest all readers of magazines. The third article is styled "*Scenes in the War of* 1812." With this article is also a map of Niagara Frontier, an illustrative cut of the interior of Fort Niagara, and the portraits of Stephen Van Rensselaer, John E. Wool, William Hamilton Merritt, and other embellishments contribute to the interests of this article. The remaining twenty articles deserve also equal attention, but we must not attempt to speak in detail of the merits of these popular magazines. They are always welcome visitors, and serve to beguile a weary hour when the harder labors of the day are done.

